# Supplementary material for: EZH2 Inhibition as New Epigenetic Treatment Option for Pancreatic Neuroendocrine Neoplasms (PanNENs)
Source: Cancers (Basel). 2021 Oct 7;13(19):5014. doi: 10.3390/cancers13195014 (PMC8508156; doi:10.3390/cancers13195014)
Supplement: Supplementary file 1 [file cancers-13-05014-s001.zip › cancers-1401279-supplementary-revised.pdf]

cancers

MDPI

Supplementary Materials

Supplementary Methods: Quantitative single cell imaging of cell death

The percentage of dead cells relative to total cell count was measured by quantitative single cell imaging of propidium- (dead cells) and Hoechst 33342-stained cells (alive and dead cells=total cells) as described previously with minor modifications [1]. BON1 and QGP1 cells were seeded at 20,000 cells/well in Ibidi optical 96-well plates (#89626, Ibidi) and incubated for 24 hours. Cells were treated in technical replicates (n=3) with vehicle control (DMSO) or indicated concentrations of GSK126. After 48 hours, cells were stained with Hoechst 33342 (5 µg/ml) and propidium iodide (50 µg/ml) at 37°C for 30 minutes, and >1000 cells/well (4-9 images) were imaged at 4x magnification using an automated fluorescence microscope (InCell 2000 Analyzer, GE Healthcare Life Sciences). Cell Profiler software (Broad Institute) was used for image segmentation and automated detection of stained nuclei [2]. The percentage of dead cells relative to vehicle control was calculated based on the ratio of PI-positive nuclei and total nuclei number.

References

1. Tousignant, K.D.; Rockstroh, A.; Poad, B.L.J.; Talebi, A.; Young, R.S.E.; Taherian Fard, A.; Gupta, R.; Zang, T.; Wang, C.; Lehman, M.L.; et al. Therapy-induced lipid uptake and remodeling underpin ferroptosis hypersensitivity in prostate cancer. *Cancer Metab.* **2020**, *8*, 11.
2. Carpenter, A.E.; Jones, T.R.; Lamprecht, M.R.; Clarke, C.; Kang, I.H.; Friman, O.; Guertin, D.A.; Chang, J.H.; Lindquist, R.A.; Moffat, J.; et al. CellProfiler: image analysis software for identifying and quantifying cell phenotypes. *Genome Biol.* **2006**, *7*, R100.

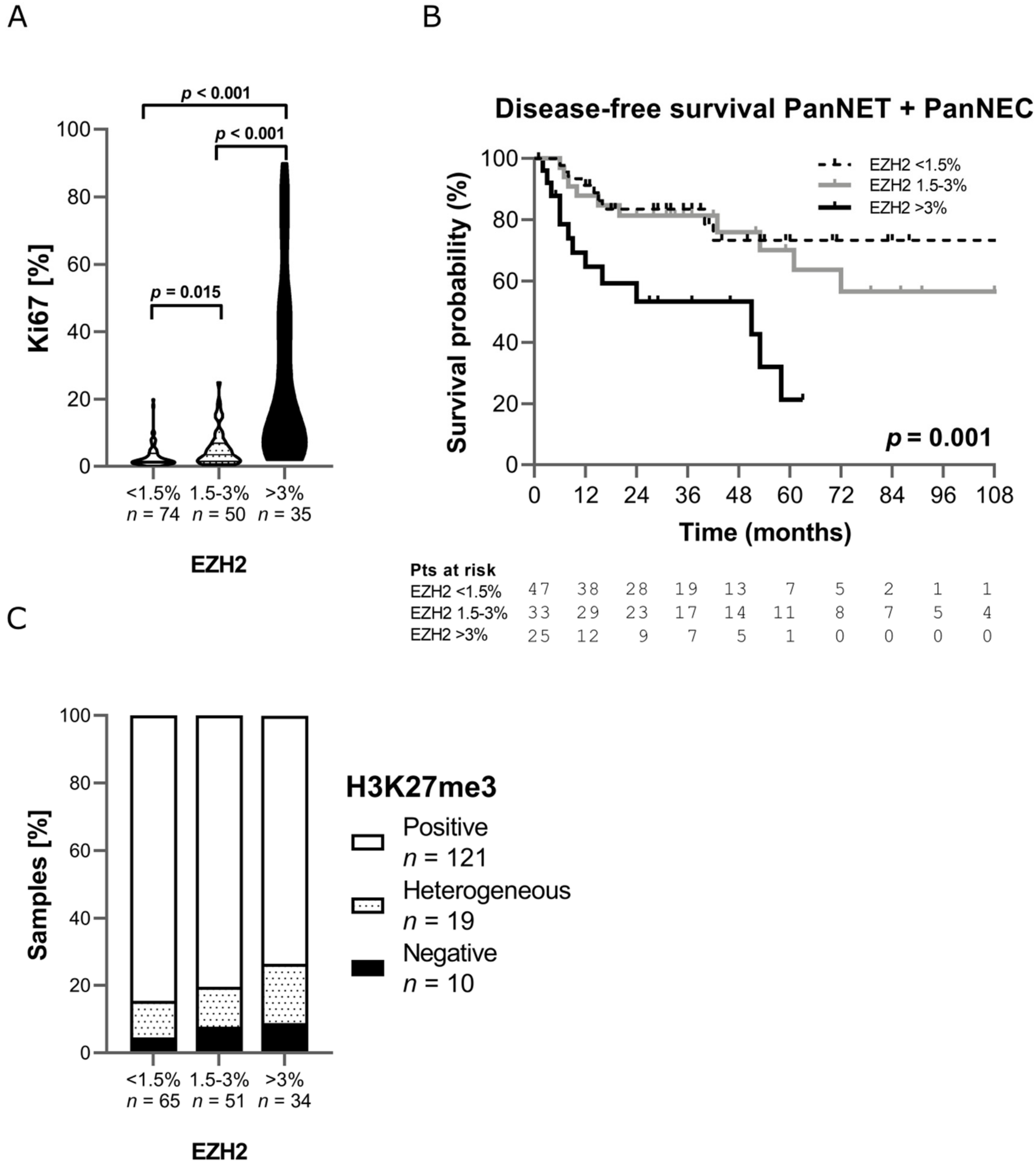

Figure S1. A) Correlation between Ki67 (%) and EZH2 (Ki67 as continuous variable available in n=159/172 patients). B) Comparison of disease-free survival between patients with low, intermediate, and high EZH2 expression including PanNECs. Patients with high EZH2 expression have significant shorter survival p=0.001. C) Correlation between H3K27me3 level and EZH2 expression in PanNEN human tissues (H3K27me3 expression available n=130/172 patients). No significant correlation was detected.

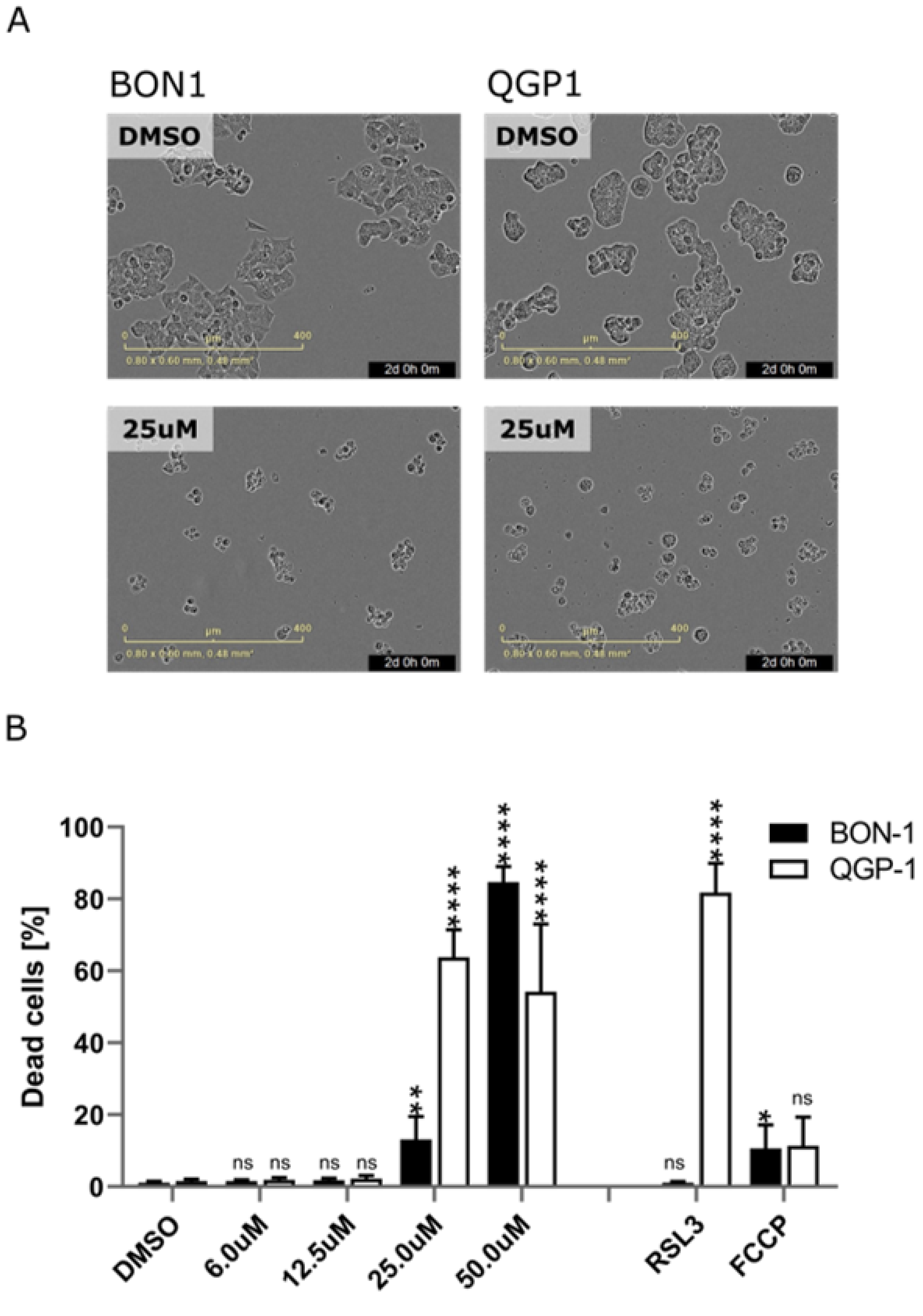

Figure S2. A) Representative images of the IncuCyte S3 imaging of BON1 and QGP1 cells after treatment with vehicle control (DMSO) and GSK126 for 48 hours. B) Graphical representation of quantitative single cell imaging of cell death of BON1 and QGP1 cells after propidium iodide (dead cells) and Hoechst 33342 staining (total cell count). Cells were treated in technical replicates (n=3 wells, >1000 cells/well) with vehicle control (DMSO) or indicated concentrations of GSK126 and incubated for 48 hours. After co-staining, replicate samples were automatically imaged with an InCell 2000 Analyzer,

analyzed by CellProfiler software, and the percentage of dead cells was calculated relative to the total cell count. A ferroptosis activator [(1S,3R)-RSL3 0.5 µM and 15 µM) and protonophore for uncoupling of the electron transport chain (Carbonyl cyanide 4-(trifluoromethoxy) phenylhydrazone, FCCP 20 µM) were used as positive controls to induced cell death.

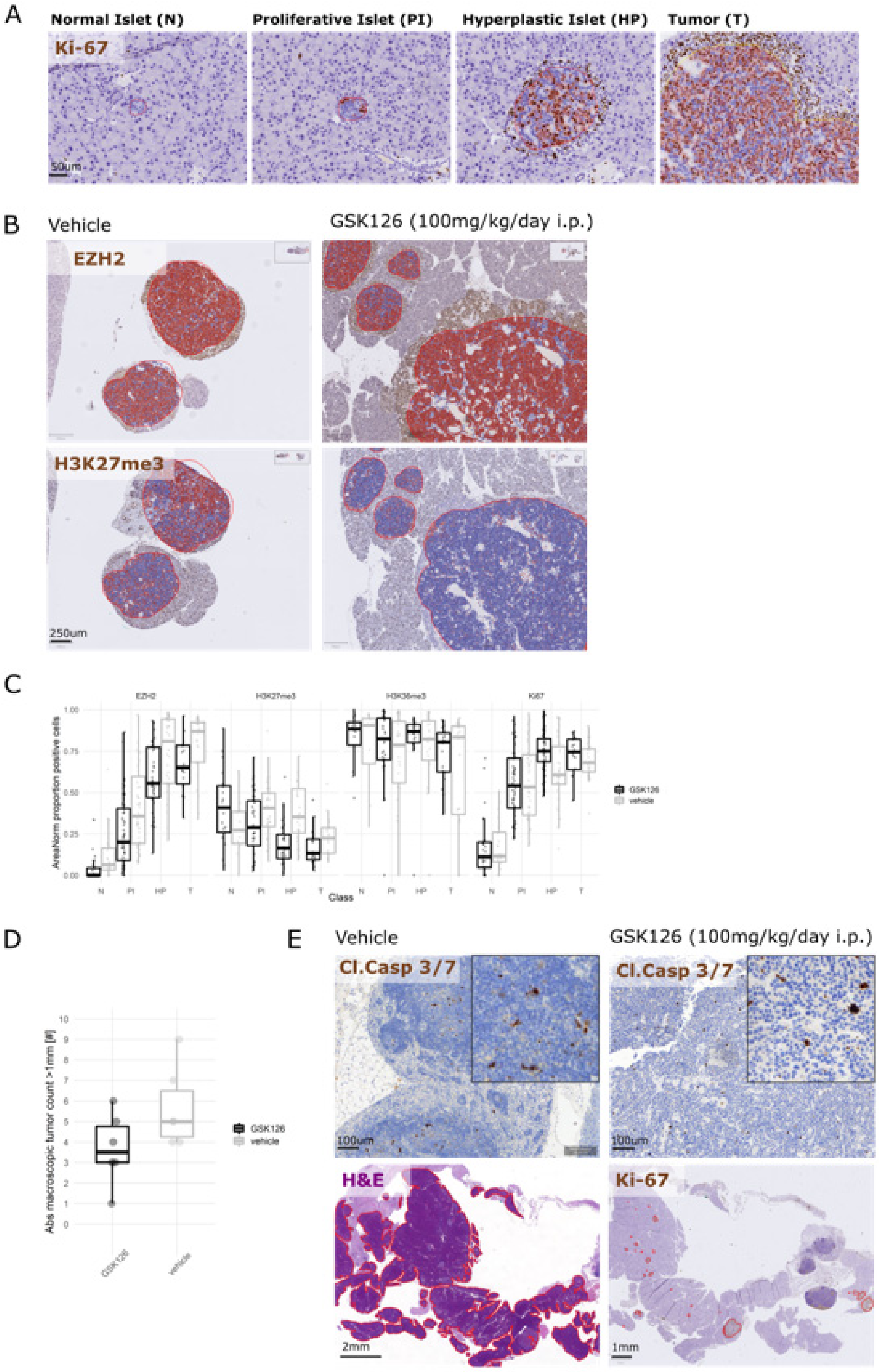

Figure S3. A) Example of tumor classification in the Rip1TAG2 model and Ki67 staining. B) EZH2 and H3K27me3 expression in control and GSK126 treated animals. While EZH2 expression does not change between treated and control mice, H3K27me3 level significantly decreased after GSK126 treatment. C) Quantification based on QuPath scoring of EZH2,

H3K27me3, H3K36me3 and Ki67 across the different tumor stages in Rip1tag2 mice GSK126 treated and control mice. D) Tumor number in GSK126 treated and control mice. E) Representative IHC for Caspase-3 and Ki67 in treated and control mice.

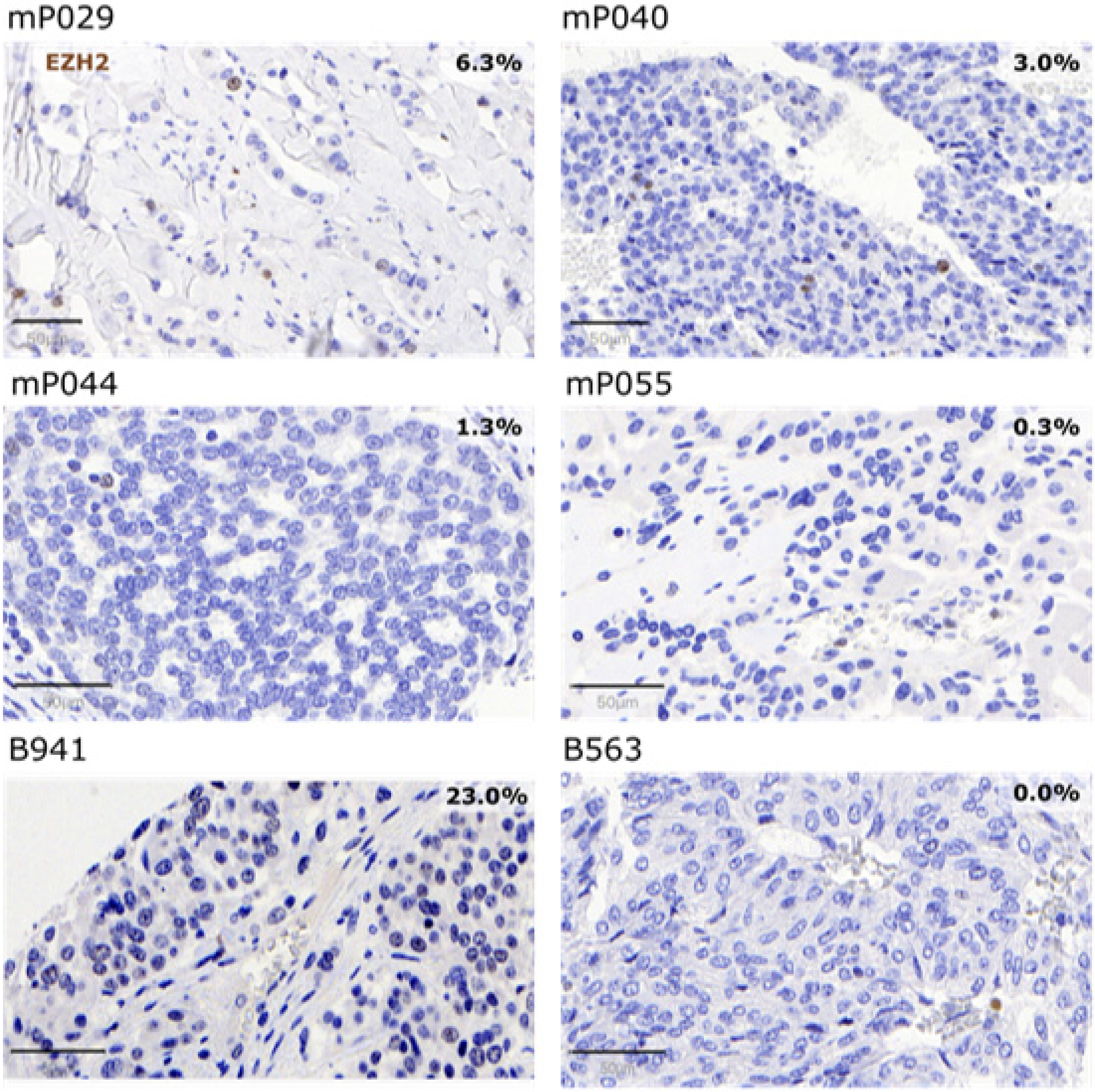

Figure S4. EZH2 immunohistochemistry on the original tumor tissue from which PanNETs tumoroids were isolated.

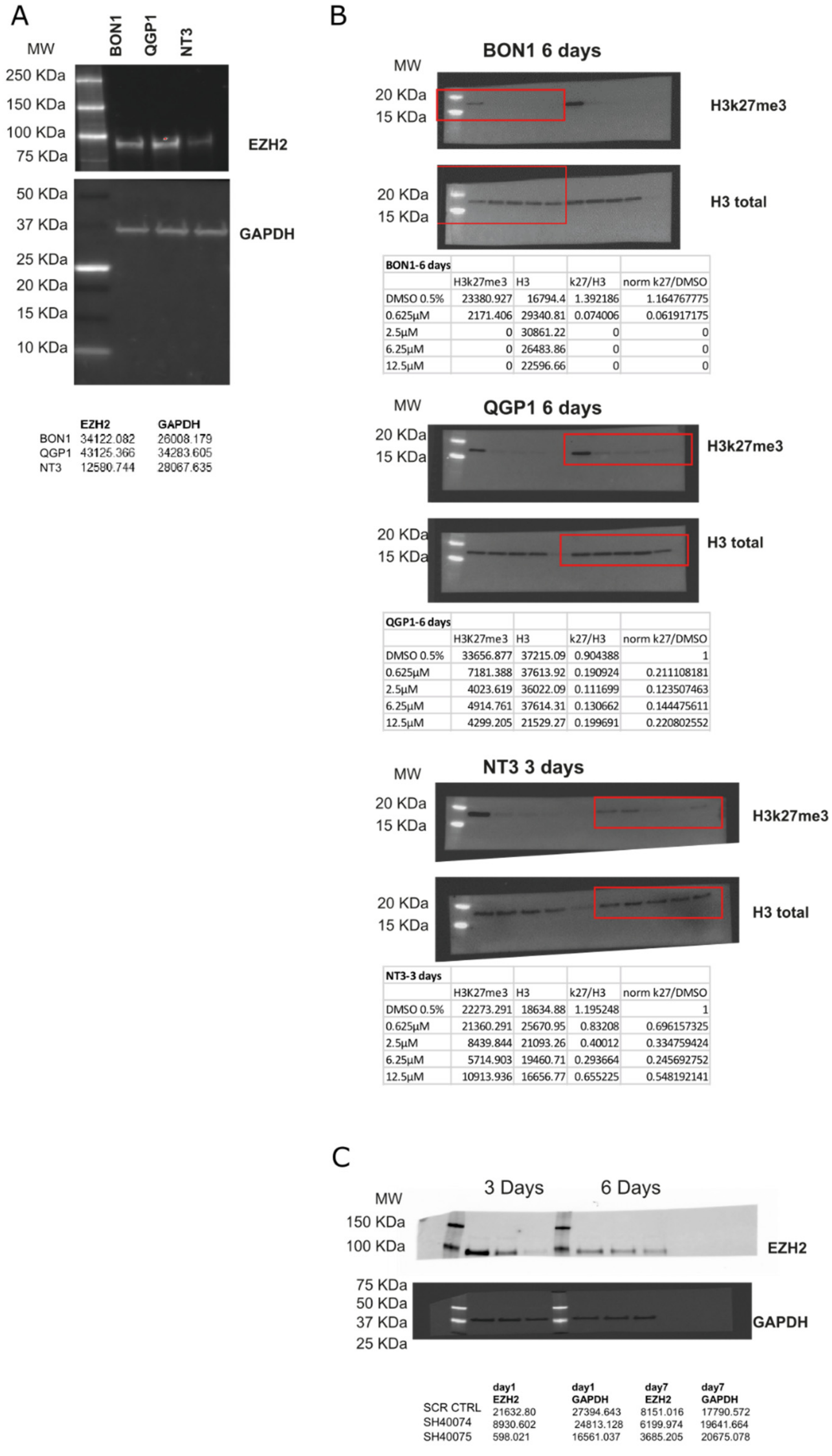

	EZH2	GAPDH
BON1	34122.082	26008.179
QGP1	43125.366	34283.605
NT3	12580.744	28067.635

BON1-6 days				
	H3k27me3	H3	k27/H3	norm k27/DMSO
DMSO 0.5%	23380.927	16794.4	1.392186	1.164767775
0.625μM	2171.406	29340.81	0.074006	0.061917175
2.5μM	0	30861.22	0	0
6.25μM	0	26483.86	0	0
12.5μM	0	22596.66	0	0

QGP1-6 days				
	H3K27me3	H3	k27/H3	norm k27/DMSO
DMSO 0.5%	33656.877	37215.09	0.904388	1
0.625μM	7181.388	37613.92	0.190924	0.211108181
2.5μM	4023.619	36022.09	0.111699	0.123507463
6.25μM	4914.761	37614.31	0.130662	0.144475611
12.5μM	4299.205	21529.27	0.199691	0.220802552

NT3-3 days				
	H3K27me3	H3	k27/H3	norm k27/DMSO
DMSO 0.5%	22273.291	18634.88	1.195248	1
0.625μM	21360.291	25670.95	0.83208	0.696157325
2.5μM	8439.844	21093.26	0.40012	0.334759424
6.25μM	5714.903	19460.71	0.293664	0.245692752
12.5μM	10913.936	16656.77	0.655225	0.548192141

	day1 EZH2	day1 GAPDH	day7 EZH2	day7 GAPDH
SCR CTRL	21632.80	27394.643	8151.016	17790.572
SH40074	8930.602	24813.128	6199.974	19641.664
SH40075	598.021	16561.037	3685.205	20675.078

Figure S5. Original western blotting membranes with the ladder Precision Plus Protein Kaleidoscope (Biorad) A) EZH2 expression level in BON1, QGP1 and NT3 cell lines (Fig 2 B). B) H3K27me3 level after 6 days of GSK126 treatment in BON1 and QGP1 and 3 days in NT3 with the correspondent band intensity measurement (fig 2D). The membrane was cut at 25 KDa. Band intensity was measured using ImageJ and area size calculation tool of plotted lane (square pixel). C) EZH2 protein level after Sh-RNA lentivirus transduction in QGP1 and correspondent GAPDH (Fig. 3A).
