# Supplementary material for: EZH2 Inhibition as New Epigenetic Treatment Option for Pancreatic Neuroendocrine Neoplasms (PanNENs)
Source: Cancers (Basel). 2021 Oct 7;13(19):5014. doi: 10.3390/cancers13195014 (PMC8508156; doi:10.3390/cancers13195014)
Supplement: Supplementary file 1 [file cancers-13-05014-s001.zip › Patient consent 1.pdf]

INSELGRUPPE

Direktion Lehre und Forschung

Etikette Insel Gruppe intern

Einwilligungserklärung zur Weiterverwendung von gesundheitsbezogenen Daten und Proben und zur Blutentnahme

Vor- und Nachname Patient/in ______________________ Geburtsdatum ______________

1) Ich stimme hiermit zu,

dass meine **gesundheitsbezogenen Daten und Proben**, die während meines ambulanten oder stationären Spitalaufenthaltes erhoben bzw. entnommen werden, der Forschung zur Verfügung stehen.

☐ Ja ☐ Nein

2) Ich stimme hiermit zu,

- dass mir zu Forschungszwecken Blut (maximal 20 Milliliter, entspricht zwei Entnahmeröhrchen) entnommen werden darf
- dass bei Bedarf zusammen mit der Blutentnahme folgende zusätzlichen Angaben erhoben werden dürfen: Grösse / Gewicht / Ethnie (Abstammung, z.B. Kaukasisch)
- dass ich mit der Nutzung von biologischem Material und gesundheitsbezogenen Daten gemäss Teil 1) einverstanden bin

☐ Ja ☐ Nein

Ich weiss, dass
- auf dem Informationsblatt (V3.0 August 2020) die Bestimmungen zur Weiterverwendung meiner Daten und Proben beschrieben sind. Ich habe diese Informationen gelesen und verstanden.
- meine persönlichen Daten geschützt sind.
- meine Daten und Proben in nationalen und internationalen Projekten, innerhalb öffentlicher und privater Institutionen, verwendet werden können.
- meine Proben zu Forschungszwecken für genetische Analysen verwendet werden können.
- ich kontaktiert werden kann, falls für mich relevante Informationen gefunden werden.
- meine Entscheidung freiwillig ist und keinen Einfluss auf meine Behandlung hat.
- meine Entscheidung zeitlich unbegrenzt gilt.
- ich meine Zustimmung jederzeit ohne Begründung zurückziehen kann.
- das Reglement der Liquid Biobank Bern jederzeit unter www.biobankbern.ch einsehbar ist.

Ort, Datum ______________ Unterschrift Patient/in, sofern urteilsfähig ______________

Ort, Datum ______________ Unterschrift gesetzlicher Vertreter, falls zutreffend (Name und Bezug zu Patient/in) ______________

Sie erhalten eine Kopie dieser Seite mit Unterschrift, wenn Sie dies wünschen. Bei Fragen wenden Sie sich bitte an Ihre behandelnden Ärzte oder an folgenden Kontakt:

Insel Gruppe AG, Direktion Lehre und Forschung CH-3010 Bern
einwilligungserklaerung-forschung@insel.ch, www.inselgruppe.ch/generalconsent
Telefon: 031 632 58 45

Information zur Weiterverwendung gesundheitsbezogener Daten und Proben zu Forschungszwecken

Sehr geehrte Patientin, sehr geehrter Patient

Die Erkennung und Behandlung von Krankheiten hat in den letzten Jahrzehnten enorme Fortschritte gemacht. Diese Entwicklung ist das Ergebnis langjähriger Forschung, an der Ärzte, Wissenschaftler und Patienten verschiedener Generationen aktiv beteiligt sind.

Die Forschung ist dabei wesentlich auf Daten aus Krankengeschichten von Patientinnen und Patienten angewiesen. Diese können zum Beispiel Ergebnisse aus Laboranalysen, Informationen zu einer Therapie oder über erbliche Veranlagungen zu bestimmten Krankheiten umfassen. Auch biologisches Material, das als Probe während eines Spitalaufenthaltes entnommen wurde und für Diagnosezwecke nicht mehr benötigt wird, ist sehr wertvoll für die Forschung. Bei diesen übriggebliebenen Proben kann es sich beispielsweise um Blut-, Urin- oder Gewebeproben handeln.

Diese Information erklärt, wie Sie als Patientin oder Patient zum medizinischen Fortschritt beitragen können und zeigt auf, wie Ihre Patientendaten und -rechte geschützt werden. Wir danken Ihnen herzlich für Ihr Interesse und Ihre Unterstützung.

Wie können Sie Ihren Beitrag zur Forschung leisten?

Wenn Sie das Einwilligungsformular mit «Ja» unterschreiben, stellen Sie Ihre gesundheitsbezogenen Daten und übriggebliebenen Proben der Forschung zur Verfügung. Die Einwilligung gilt für alle Daten, die bereits im Spital erhoben wurden oder zukünftig erhoben werden. Dies gilt im gleichen Sinne für die Proben.

Ihre Einwilligung ist freiwillig. Sie gilt zeitlich unbegrenzt, falls sie nicht widerrufen (zurückgezogen) wird. Sie können Ihre Einwilligung jederzeit über die untenstehende Kontaktadresse widerrufen, ohne eine Begründung dafür abzugeben. Nach dem Widerruf werden Ihre Daten und Proben für neue Forschungsprojekte nicht mehr zur Verfügung gestellt. Ihre Entscheidung hat keinen Einfluss auf Ihre medizinische Behandlung.

Wie werden Ihre gesundheitsbezogenen Daten und Proben geschützt?

Ihre Daten werden im Spital gemäss den gesetzlichen Vorschriften bearbeitet und geschützt. Nur befugte Mitarbeiter des Spitals, wie beispielsweise Ärzte, haben Einsicht in die unverschlüsselten Daten aus Ihrer Krankengeschichte und Zugang zu Ihren unverschlüsselten Proben. Ihre Proben sind in Biobanken verwahrt. Eine Biobank stellt eine strukturierte Sammlung verschiedener Proben unter bestimmten Sicherheitsbestimmungen (Biobankreglement) dar.

Wenn Ihre Daten und Proben für Forschungsprojekte verwendet werden, so werden diese verschlüsselt oder anonymisiert. Verschlüsselt bedeutet, dass alle personenbezogenen Daten wie Ihr Name oder Geburtsdatum durch einen Code ersetzt werden. Der Schlüssel, der zeigt, welcher Code zu welcher Person gehört, wird durch eine nicht am Forschungsprojekt beteiligte Person sicher verwahrt. Personen, die keinen Zugang zum Schlüssel haben, können Sie nicht identifizieren. Werden Daten und Proben anonymisiert, gibt es keinen Schlüssel, der auf Ihre Person zurückführt.

Wer darf Ihre gesundheitsbezogenen Daten und Proben verwenden?

Ihre Daten und Proben werden berechtigten Forschenden an unserem Spital für Forschungsprojekte zur Verfügung gestellt oder können in Forschungsprojekten in Zusammenarbeit mit anderen öffentlichen oder privaten Institutionen verwendet werden (andere Spitäler, Universitäten oder pharmazeutische Unternehmen). Die Projekte können in der Schweiz oder im Ausland durchgeführt werden und gegebenenfalls genetische Analysen beinhalten. Bei Forschungsprojekten im Ausland gelten mindestens die gleichen Anforderungen an den Datenschutz wie in der Schweiz. Forschungsprojekte unterliegen generell einer Überprüfung der zuständigen Ethikkommission.

Werden Sie über Forschungsresultate informiert?

Forschungsprojekte mit Daten und Proben führen in der Regel nicht zu Informationen, die unmittelbar für die Gesundheit einer einzelnen Person von Belang sind. Sollte dennoch ein für Sie bedeutendes Ergebnis gefunden werden und eine medizinische Massnahme verfügbar sein, ist eine Kontaktaufnahme durch das Spital möglich.

Werden Sie finanzielle Vor- oder Nachteile haben?

Es entstehen für Sie keine zusätzlichen Kosten. Es ist von Gesetzes wegen ausgeschlossen, mit Ihren Daten und Proben Geld zu erwirtschaften. Es entsteht daher weder für Sie noch für das Spital ein finanzieller Vorteil.

Falls Sie noch Fragen haben oder zusätzliche Informationen wünschen, wenden Sie sich bitte an die untenstehende Kontaktadresse oder besuchen Sie unsere Website.

Information zu einer Blutentnahme für die Liquid BioBank Bern

Für aussagekräftige Forschungsergebnisse sind Forschende darauf angewiesen, auf zahlreiche qualitativ hochwertige Proben zurückgreifen zu können. Aus diesem Grund ist es für die Insel Gruppe ausgesprochen wichtig von den Patienten eine zusätzliche Blutprobe zu entnehmen. Ihre Einwilligung zu dieser Blutentnahme geben Sie gesondert.

Wie wird Ihre Probe genutzt?

Für die Aufbewahrung und Weitergabe Ihrer Probe gelten dieselben Vorschriften wie für die gesundheitsbezogenen Informationen und medizinischen Proben. Insbesondere können Sie die Einwilligung jederzeit ohne Begründung verweigern oder widerrufen. Zusätzlich ist die Verwaltung und Weitergabe der Proben in einem von der kantonalen Ethikkommission bewilligten Reglement definiert.

Warum wird eine zusätzliche Blutentnahme benötigt?

Im Vergleich zu Untersuchungen, die für Ihre Behandlung durchgeführt werden, hat die Forschung andere Anforderungen an die Probenqualität. Insbesondere ist wichtig, dass alle in einer Studie verwendeten Proben auf die gleiche Art verarbeitet wurden.
Entnahme, Transport und Lagerung beeinflussen die Qualität einer Blutprobe. Dank der zusätzlichen Blutentnahme können diese Prozesse standardisiert und dokumentiert werden. Diese Qualitätssicherung und Lagerung werden in der Liquid BioBank Bern sichergestellt. Die Liquid BioBank Bern kann dadurch den verschiedenen Forschungsvorhaben Proben mit einheitlicher und gleichbleibender Qualität zur Verfügung stellen.

Warum sind zusätzliche Angaben (Grösse/Gewicht/Ethnie) erforderlich?

Ihre Blutprobe ist für die Forschung äusserst wertvoll. Neben den bereits vorhandenen gesundheitlichen Daten dienen die zusätzlichen Angaben dazu, Proben zu kategorisieren. So kann Ihre Probe denjenigen Forschungsvorhaben zur Verfügung gestellt werden, welche den grössten Nutzen daraus ziehen können.

Wann erfolgt die Blutentnahme?

Die Blutentnahme erfolgt im Rahmen Ihres Spitalaufenthaltes. Sie werden nicht speziell für eine BioBank-Blutentnahme eingeladen.

Was ist eine Biobank?

In einer sogenannten Biobank werden Proben gesammelt, die im Rahmen von Studien oder für die Forschung allgemein, angelegt werden. Zu jeder eingelagerten Probe werden Daten zur Probe und zum Spender selbst gespeichert.

Welchen Zweck verfolgt die Liquid BioBank Bern?

In den letzten Jahren wurden Proben meist gezielt für einzelne Studien gesammelt. Wertvolles Material von Probanden stand somit nur einem beschränkten Kreis von Forschenden zur Verfügung. Mit dem Aufbau der Liquid BioBank Bern sollen Proben gesammelt und verschiedenen Forschungsprojekten zur Verfügung gestellt werden. Dadurch wird der Nutzen von einzelnen Proben für die Forschung erhöht und der Aufwand für die medizinische Forschung gesenkt.

Wo finden Sie weitere Informationen zur Liquid BioBank Bern?

Die Liquid BioBank Bern (LBB) gehört der Insel Gruppe. Die LBB wurde eingerichtet, um insbesondere biomedizinische Forschung nachhaltig zu fördern und durch Zurverfügungstellung von Patientenproben mit im internationalen Vergleich herausragender Qualität zu unterstützen. Das Ziel ist es Prädiktion, Prävention, Diagnose und Therapie in der Humanmedizin zu verbessern. Ausserdem können mit den gesammelten Proben und Daten auch grundlegende Mechanismen zum Verständnis von Krankheiten erforscht werden.

Wenn Sie mehr über die Liquid BioBank Bern erfahren möchten, besuchen Sie unsere Website
www.biobankbern.ch
