# Supplementary material for: EZH2 Inhibition as New Epigenetic Treatment Option for Pancreatic Neuroendocrine Neoplasms (PanNENs)
Source: Cancers (Basel). 2021 Oct 7;13(19):5014. doi: 10.3390/cancers13195014 (PMC8508156; doi:10.3390/cancers13195014)
Supplement: Supplementary file 1 [file cancers-13-05014-s001.zip › Patient consent 2.pdf]

PREMESSA

Il Comitato Etico per l'assistenza e per la ricerca dell'Ospedale San Raffaele di Milano, Istituto di Ricovero e Cura a Carattere Scientifico, istituito con atto deliberativo del Consiglio di Amministrazione della Fondazione Centro San Raffaele del Monte Tabor in data 24 aprile 1986 e ricostituito in base al Decreto Ministeriale del 12 maggio 2006 in data 15 dicembre 2006, è un organismo indipendente ai sensi delle vigenti disposizioni comunitarie e interne e si ispira tutt'ora ai principi che hanno informato lo Statuto della Fondazione Centro San Raffaele del Monte Tabor, in particolare la tutela del malato nella sua dignità di persona.
La progressione della ricerca in ambito sanitario è strettamente correlata alla disponibilità di materiale biologico da poter utilizzare. Poiché esso costituisce un'importante fonte di risorse per la diagnosi e per la ricerca scientifica, è di fondamentale importanza disporre di un numero sempre maggiore di campioni biologici che dovranno essere raccolti, conservati ed utilizzati secondo i precetti normativi nazionali (art.32 della Costituzione.) ed internazionali ("Convenzione di Oviedo" e la "Carta dei diritti fondamentali dell'Unione Europea, 2000/C 364/01") e secondo i principi sopra enunciati. A tal proposito, a garanzia dell'osservanza dei principi normativi ed istituzionali, ogni singola ricerca e/o sperimentazione su materiale biologico è subordinata all'approvazione del Comitato Etico OSR, cui deve essere fornita completa documentazione e informazione, con particolare riguardo al razionale, allo scopo, alla novità e al valore dello studio, al disegno sperimentale, alla dettagliata descrizione delle procedure e delle metodologie che saranno utilizzate, all'analisi dei rischi e dei benefici per i soggetti coinvolti e la comunità.
Devono essere altresì indicate le modalità per una conservazione sicura dei campioni e dei dati raccolti o generati, nonché le modalità intese a tutelarne la riservatezza.

***

Per regolamentare la raccolta, la conservazione e l'utilizzo di materiale biologico umano, l'Ospedale San Raffaele ha costituito una **BANCA BIOLOGICA**, che, secondo la Raccomandazione del Consiglio d'Europa, R(94)1, del 14.3.1994, è definita come un'organizzazione non-profit ufficialmente riconosciuta dalle Autorità Sanitarie competenti, che deve garantire il trattamento, la conservazione e la distribuzione del materiale biologico.
La banca biologica deve essere gestita secondo il seguente regolamento:

1. E' vietata qualsiasi commercializzazione o utilizzazione a scopo di lucro diretto del materiale biologico "**donato**" dal soggetto.

2.
a) Il materiale biologico viene acquisito, conservato in una banca biologica e utilizzato per fini di ricerca o di sperimentazione solo previo consenso scritto, libero ed informato della persona da cui il materiale proviene. Pertanto i campioni biologici prelevati e i dati genetici raccolti per scopi di tutela della salute possono essere conservati ed utilizzati per finalità di ricerca scientifica o statistica con il consenso informato delle persone interessate, eccetto che nei casi

di indagini statistiche o ricerche scientifiche previste dalla legge o per necessità di sanità pubblica superiori rispetto ai diritti individuali.
Qualora il soggetto sia deceduto senza aver potuto esprimere il consenso esplicito al prelievo di tessuti, questo può essere anche presunto (o espresso dai familiari) purché il medico responsabile si sia preventivamente accertato presso i familiari del defunto sull'esistenza di eventuali pareri contrari di quest'ultimo o nel caso abbia fatto obiezioni sul prelievo.

b) Nel caso della conservazione e dell'ulteriore utilizzo di campioni biologici e di dati genetici raccolti per la realizzazione di progetti di ricerca e indagini statistiche, diversi da quelli per i quali è stato originariamente acquisito il consenso informato, sono consentiti limitatamente al perseguimento di scopi scientifici e statistici collegati con quelli originari. Qualora i campioni biologici ed i dati genetici ad essi correlati non consentano più di identificare l'interessato o a causa di particolari ragioni non sia possibile informarlo, malgrado sia stato compiuto ogni ragionevole sforzo per raggiungerlo, il programma di ricerca deve essere oggetto di motivato parere favorevole del competente Comitato Etico ed autorizzato appositamente dal Garante ai sensi dell'art. 110 del Codice della Privacy. La garanzia degli scopi scientifici, per progetti derivanti dall'utilizzo del materiale biologico donato ai fini di ricerca o di sperimentazione futura e non programmata, è data dal parere espresso dal Comitato Etico dell'Ospedale San Raffaele.

c) Il prelievo da persona non capace può essere effettuato solo quando assolutamente indispensabile, sulla base del consenso informato prestato da chi lo rappresenti. Non si procederà comunque al prelievo in caso di dissenso della persona interessata o dei prossimi congiunti. Dopo il raggiungimento della maggiore età l'informativa è fornita all'interessato anche ai fini dell'acquisizione di un nuovo consenso quando questo è necessario.

d) Queste disposizioni si applicano anche alla utilizzazione per fini di ricerca e/o di sperimentazione di materiali biologici prelevati per motivi diagnostici o chirurgici.

e) I campioni biologici prelevati e i dati genetici trattati per l'esecuzione di test e di screening genetici sono conservati per un periodo di tempo non superiore a quello necessario allo svolgimento delle analisi o al perseguimento degli scopi per i quali sono stati raccolti o successivamente utilizzati a meno che il paziente non abbia consentito la conservazione del reperto o di dati genetici per una durata illimitata alle condizioni sopra indicate.

f) Resta salva la possibilità di brevettare ogni eventuale invenzione derivata o connessa alla ricerca effettuata (e/o sperimentazione) nel pieno rispetto delle vigenti norme di legge, così come anche indicato nel modello di consenso informato.

3. Il materiale biologico deve essere codificato, in modo che non sia possibile ad alcuno risalire alla persona che ne ha fatto donazione, tranne ai responsabili della ricerca o della sperimentazione e della banca biologica, i quali potranno accedere a tali informazioni solo in vista di un beneficio del donatore.

4. Non è autorizzata alcuna utilizzazione attuale o futura di materiale biologico, comunque motivata, per obiettivi incompatibili con i principi etici richiamati in premessa.

5.
a) Il trasferimento ad altri Enti di materiale biologico conservato presso la Banca Biologica dell'Ospedale è ammesso.

b) Esso è possibile solo nel caso in cui gli Enti destinatari perseguano obiettivi analoghi a quelli dell'Istituto Scientifico San Raffaele in conformità ai suoi principi che dovranno essere dichiarati per iscritto dall'Ente ricevente.

c) Ogni trasferimento di materiale biologico a Enti diversi dall'Istituto Scientifico San Raffaele, nonché ogni singola ricerca e/o sperimentazione sul medesimo materiale che sia da effettuarsi presso uno di tali Enti, è subordinata all'approvazione del Comitato Etico OSR cui deve essere fornita completa documentazione ed informazione.

d) Il materiale biologico trasferito non potrà essere ulteriormente trasferito o reso in qualsiasi modo disponibile, né interamente né in parte, a destinatari che non siano indicati nel documento di approvazione del Comitato Etico OSR.

6. Il presente regolamento deve essere reso noto a tutti coloro ai quali si richiede il consenso per il prelievo, l'utilizzazione e il deposito di materiale biologico e deve essere allegato al modulo di consenso informato.

Letto, per accettazione

____

(Firma Responsabile Banca Biologica)

INFORMATIVA PER IL PRELIEVO DI MATERIALE BIOLOGICO DA DONARE AI FINI DI RICERCA SCIENTIFICA

Gentile Signora, Egregio Signore,

diverse esperienze di istituti italiani e internazionali, incluso il nostro Istituto, hanno permesso di constatare l'importanza di costituire una Banca Biologica al fine di conservare materiali organici derivanti dal corpo umano (ad esempio, sangue, urine, tessuti, etc.). Il materiale biologico è una fonte insostituibile per la ricerca scientifica: lo studio e la sperimentazione su tale materiale offre preziose informazioni a fini terapeutici per malattie ancora incurabili.

Con il presente modulo, intendiamo informarLa affinché possa rilasciare un consenso - libero e consapevole - al prelievo di materiale biologico, al suo utilizzo ed alla sua eventuale conservazione, per esclusivi fini di ricerca scientifica. Le chiediamo, pertanto, di leggere attentamente questa informativa e, all'occorrenza, di chiedere tutte le delucidazioni di cui avesse bisogno.

Il prelievo di tessuto o materiale biologico, previo il Suo consenso, potrà avvenire sia in regime ambulatoriale che in regime di ricovero. In quest'ultimo caso, qualora debba sottoporsi ad un intervento chirurgico, La informiamo che il materiale prelevato sarà unicamente quello oggetto dell'intervento chirurgico o piccole eccedenze rispetto alla parte anatomica da asportare

Il materiale raccolto verrà utilizzato solo ed unicamente per progetti di ricerca scientifica condotti dal nostro Istituto e preventivamente approvati dal nostro Comitato Etico. In particolare, il campione biologico che Lei andrebbe a "donare" sarà utilizzato per futuri progetti di ricerca scientifica il cui scopo sarà lo studio dei tumori endocrini, ed in particolare la valutazione della loro "firma biologica". Con firma biologica intendiamo la produzione da parte del tumore di marcatori valutabili nei campioni biologici dei soggetti affetti, che potranno in futuro, aiutare noi clinici nella diagnosi, nella determinazione della aggressività di malattia e nella determinazione di trattamenti appropriati e personalizzati per chi, come Lei, è affetto da tumore neuroendocrino.

Con il Suo consenso, eventuali rimanenze verranno conservate presso la nostra Banca Biologica che ne garantirà la custodia e l'utilizzo per un periodo illimitato. Contrariamente, la parte eccedente di materiale biologico verrà distrutta al termine del Progetto di ricerca per il quale era avvenuto il prelievo..

Tuttavia, considerato il vantaggio che potrebbe trarre la scienza, l'umanità intera e, di conseguenza, gli ammalati nel disporre di reperti così preziosi che costituiscono la "materia prima" per chi dedica la propria vita a scoprire nuove malattie e studiarne le modalità per

sconfiggerle, le chiediamo di "donarci" il Suo reperto affinché il nostro Istituto possa archiviarlo nella propria Banca Biologica e conservarlo per periodo illimitato utilizzandolo in futuri progetti di ricerca ad oggi non individuabili.
In caso di programmi di ricerca che non siano correlati con quello per il quale è stato prelevato il materiale biologico, verrà profuso ogni ragionevole sforzo per chiederLe un nuovo consenso al suo utilizzo.

Le assicuriamo che è vietata qualsiasi commercializzazione o utilizzazione a scopo di lucro del materiale biologico prelevato, mentre resta la facoltà per il nostro Istituto, o per il patrocinatore della ricerca, di brevettare ogni eventuale invenzione derivata o connessa all'attività di ricerca scientifica svolta, per fini diagnostici o terapeutici nel pieno rispetto delle vigenti norme di legge. Gli eventuali proventi derivanti da tale attività verranno reinvestiti nella ricerca.

Il prelievo non Le arrecherà alcun danno oltre al minimo rischio legato al posizionamento del butterfly ed ai prelievi di sangue venoso.

I risultati derivanti dagli studi condotti sul materiale biologico da Lei fornito saranno trattati in maniera tale da conservarne scrupolosamente la riservatezza.

Dalla donazione dei suoi campioni biologici non ci sono benefici diretti per Lei, però, ci consentirà di acquisire maggiori conoscenze scientifiche da utilizzare in futuro per migliorare gli approcci terapeutici per i pazienti che si troveranno nella sua stessa condizione clinica.

Lei è libero/a di non donare il suoi campioni biologici. In questo caso, comunque, i medici continueranno a seguirla con la dovuta attenzione. La sua decisione non influenzerà in alcun modo la cura e l'attenzione che i medici e tutto il personale Le dedicherà
In qualsiasi momento Lei può ritirare l'autorizzazione alla conservazione o all'utilizzazione del materiale prelevato, nel qual caso, qualora il Suo materiale biologico sia ancora individuabile, verrà completamente distrutto a norma di legge.
Per ulteriori informazioni e comunicazioni durante lo studio sarà a disposizione il seguente personale:

- Responsabile dello studio: Dr. Marco F. Manzoni. manzoni.marco@hsr.it. 02.2643.3180
- Collaboratore di riferimento: Dr.ssa Valentina Villa, villa.valentina@hsr.it 02.2643.5262
- Collaboratore di riferimento: Dr.ssa Dolcetta Capuzzo Anna, dolcettacapuzzo.anna@hsr.it

CONSENSO INFORMATO

Il sottoscritto/a…………………………………….. nato a ...., il .... , residente in ....

dichiara

di acconsentire alla donazione del materiale biologico che gli/le verrà prelevato ed alla sua conservazione presso la Banca Biologica dell'Ospedale San Raffaele.

dichiara

di essere stato esaurientemente informato sui motivi e sui rischi dell'operazione di prelievo e che gli/le è stato comunicato che tutti i dati personali raccolti saranno trattati secondo le modalità previste dal D.Lgs. 196/03 e, comunque, secondo le modalità che verranno illustrate con un separato atto.

Data ……………Firma ………………………………………………………………………..

Data……………Firma del medico che ha raccolto il consenso …………………………………

IL PRESENTE MODULO DI CONSENSO SI INTENDE COMPLETATO CON QUANTO STABILITO DALLE LINEE GUIDA REDATTE DAL COMITATO NAZIONALE DI BIOETICA (FAC SIMILE PRESENTE A PAG. 26 TESTO DISPONIBILE SU SITO WEB DEL LA PRESIDENZA DEL CONSIGLIO DEI MINISTRI)
http://www.governo.it/biotecnologie/documenti/Consenso_Informato_allegato_Petrini_2009.pdf

INFORMATIVA
ai sensi dell'art.13 del D.Lgs.196/03
(Codice in materia di protezione dei dati personali)

****

Gentile Signora, Egregio Signore,

come sicuramente saprà, per sconfiggere tutte le malattie è necessario un grande impegno da parte dei ricercatori, sono necessarie parecchie risorse economiche ma, soprattutto, è necessario disporre di un vasto numero di campioni biologici che costituiscono un'importante fonte di risorse per la diagnosi e per la ricerca.

Per questo motivo, affinché possa liberamente decidere se consentirci la raccolta, l'utilizzo e la conservazione del Suo campione biologico e dei Suoi dati personali (tra cui anche quelli genetici), La informiamo come il nostro Istituto andrà ad effettuare l'eventuale trattamento.

TITOLARI E FINALITA' DEL TRATTAMENTO, NATURA DEI DATI:

L'Ospedale San Raffaele, con sede in Milano, in via Olgettina n.60 tratterà i Suoi dati personali (tra cui anche i suoi dati genetici, ossia i dati che riguardano la costituzione genotipica, se raccolti) ed utilizzeranno i Suoi campioni biologici (campioni di sangue o di tessuto) nella misura in cui siano indispensabili alla realizzazione del Progetto di ricerca denominato Biobanca Tumori Neuroendocrini: NETBANK001

Il trattamento dei suddetti dati e dei suddetti campioni, qualora autorizzato, sarà indispensabile allo svolgimento del suddetto progetto e, qualora Lei lo consenta, anche per la realizzazione di ulteriori progetti di ricerca futuri, ad oggi non identificabili.

MODALITÀ DI TRATTAMENTO, MISURE DI SICUREZZA E CONSERVAZIONE

I suoi dati personali (tra cui eventualmente quelli genetici, ossia i dati che riguardano la costituzione genotipica) saranno raccolti, gestiti e custoditi sia in formato cartaceo che elettronico.

Il medico che condurrà il progetto di ricerca identificherà i Suoi dati con un codice; soltanto il medico ed il suo team, debitamente autorizzato, potranno collegare questo codice al Suo nominativo.

Ogni reperto biologico verrà conservato mediante l'utilizzazione di codici identificativi che renderanno individuabile il donatore solo in caso di necessità.

Sia i campioni biologici che tutti i dati sensibili che La riguardano, compreso eventuali dati genetici, saranno raccolti, gestiti e conservati dal titolare secondo idonee misure di sicurezza, così come previsto dall'Allegato B del D.Lgs.196/03.

I campioni biologici verranno custoditi nella Banca Biologica di questo Istituto secondo i massimi criteri di sicurezza adottabili per prevenirne la trafugazione, l'utilizzo illecito e non autorizzato, nonché la distruzione accidentale o per eventi avversi.

Allo stesso modo i dati che La riguardano, compresi quelli relativi all'identità genetica, verranno trattati esclusivamente all'interno di locali protetti, accessibili ai soli "incaricati" dei trattamenti appositamente autorizzati. Il trasporto del materiale biologico all'esterno dei locali riservati al loro trattamento avverrà in contenitori muniti di serratura o dispositivi equipollenti; l'eventuale trasferimento dei dati in formato elettronico a persone autorizzate sarà esclusivamente cifrato.

La conservazione dei dati e/o dei campioni biologici sarà limitato alla realizzazione del progetto di ricerca per cui sono stati raccolti. Qualora Lei abbia deciso - in maniera del tutto autonoma e libera da condizionamenti - di "donare" a questo Ospedale il campione biologico prelevato, questo sarà utilizzato, assieme ai Suoi dati, per progetti futuri di ricerca ad oggi non individuabili ma che saranno garantiti, dal punto di vista etico e della riservatezza, da un apposito parere espresso preventivamente dal Comitato Etico di questo Istituto.

COMUNICAZIONE A TERZI

Sia i suddetti dati che i campioni biologici potranno essere comunicati e trasferiti, su espressa richiesta, ad Aziende farmaceutiche o ad enti di ricerca per i quali la conoscenza dei suddetti dati risulti necessaria o comunque funzionale allo svolgimento di progetti di ricerca congiunti con quelli svolti dallo scrivente Istituto. In questo caso i dati verranno comunicati in maniera codificata. Nel caso in cui, invece, i progetti di ricerca vengano svolti in maniera autonoma dalle società farmaceutiche o degli enti di ricerca richiedenti le informazioni inviate saranno rese completamente anonime.

Le suddette società potranno risiedere anche in Paesi non appartenenti all'Unione Europea che non garantiscono un adeguato livello di protezione dei dati personali.

I Suoi dati saranno diffusi solo in forma rigorosamente anonima in occasione di convegni scientifici o attraverso pubblicazioni scientifiche o statistiche.

ESERCIZIO DEI DIRITTI

Ai sensi e per gli effetti dell'articolo 7 del D.Lgs. 196/03, rivolgendosi direttamente all'Ospedale San Raffaele, Lei ha diritto di ottenere:
a) la conferma dell'esistenza o meno di dati personali che La riguardano, anche se non ancora registrati, e la loro comunicazione in forma intelligibile.
b) l'aggiornamento, la rettificazione ovvero, quando vi ha interesse, l'integrazione dei dati;
c) la cancellazione, la trasformazione in forma anonima o il blocco dei dati trattati in violazione di legge, compresi quelli di cui non è necessaria la conservazione in relazione agli scopi per i quali i dati sono stati raccolti o successivamente trattati;

d) l'attestazione che le operazioni di cui alle lettere b) e c) sono state portate a conoscenza, anche per quanto riguarda il loro contenuto, di coloro ai quali i dati sono stati comunicati o diffusi, eccettuato il caso in cui tale adempimento si rivela impossibile o comporta un impiego di mezzi manifestamente sproporzionato rispetto al diritto tutelato.

Lei ha diritto di opporsi, in tutto o in parte, per motivi legittimi al trattamento dei dati personali che La riguardano, ancorché pertinenti allo scopo della raccolta.

Potrà revocare il consenso in ogni momento e senza fornire alcuna giustificazione; in tal caso, i campioni biologici a Lei correlati, qualora ancora individuabili, verranno completamente distrutti. Non saranno raccolti ulteriori dati che La riguardano ferma restando l'utilizzazione di quelli eventualmente già raccolti per determinare, senza alterarli, i risultati della ricerca.

TITOLARI E RESPONSABILI DEL TRATTAMENTO

L'Ospedale San Raffaele, con sede in Milano, in via Olgettina n.60 ricopre il ruolo di Titolare del trattamento dei dati raccolti.

Per i dati ed i campioni biologici raccolti ai fini di ricerca, l'Ospedale ha nominato Responsabili del trattamento i direttori di dipartimento clinico, i direttori delle divisioni di ricerca, nonché il direttore operativo di quest'ultima, i cui nominativi sono depositati presso l'Ufficio Legale dell'Ospedale, ai sensi dell'art.29 del D.Lgs.196/03, rispettivamente:

Direttore di dipartimento clinico: Prof. Bosi Emanuele

Direttore delle divisioni di ricerca: Prof. Bosi Emanuele

Direttore operativo di ricerca: Dr. Manzoni Marco Federico

CONSENSO

Io sottoscritto ....nato a ....

il ....e residente a ....in via ....

tel ……………………..

letta l'informativa sopra riportata ed avendone compreso l'intero contenuto, con la sottoscrizione della presente consento il trattamento dei miei dati personali compresi quelli di natura genetica e l'utilizzo dei miei campioni biologici per i progetti di ricerca menzionato nell'informativa.
□ SI □ NO

____

Acconsento alla comunicazione dei suddetti dati a enti di ricerca o aziende farmaceutiche direttamente impegnate nel progetto o che partecipano a studi ad esso connessi.
□ SI □ NO

____

Acconsento al trasferimento dei suddetti dati al di fuori dell'Unione Europea per gli scopi della ricerca nei limiti e con le modalità indicate nell' informativa.
□ SI □ NO

____

Acconsento che mi vengano comunicate eventuali notizie che mi riguardino, qualora quest'ultime rappresentino per il sottoscritto a parere del personale addetto allo studio, un beneficio concreto e diretto in termini di terapia, prevenzione o di consapevolezza delle scelte riproduttive;
□ SI □ NO

____

Autorizzo la conservazione dei suddetti dati e dei campioni biologici nella banca biologica dell'Ospedale, per scopi di ricerca scientifica futuri ad oggi non identificabili;
□ SI □ NO

Data ________________ NOME E COGNOME DELL'INTERESSATO

____
(in stampatello)

____
